# Publisher Correction: Long-range spatio-temporal correlations in multimode fibers for pulse delivery

**DOI:** 10.1038/s41467-020-18909-4

**Published:** 2020-09-30

**Authors:** Wen Xiong, Chia Wei Hsu, Hui Cao

**Affiliations:** Department of Applied Physics, Yale University, New Haven, CT 06520 USA

**Keywords:** Adaptive optics, Fibre optics and optical communications

Correction to: *Nature Communications* 10.1038/s41467-019-10916-4, published online 5 July 2019.

The original version of this Article contained an error in the second paragraph of the “Static and dynamic correlations” section of the “Results” and incorrectly read “–Δ*I* (*N*−1)”. The corrected version reads “–Δ*I/* (*N*−1)”.

This error has been corrected in both the PDF and HTML versions of the article.

