# Supplementary material for: Long-range spatio-temporal correlations in multimode fibers for pulse delivery
Source: Nat Commun. 2019 Jul 5;10:2973. doi: 10.1038/s41467-019-10916-4 (PMC6611823; doi:10.1038/s41467-019-10916-4)
Supplement: Supplementary file 1 — Supplementary Information [file 41467_2019_10916_MOESM1_ESM.pdf]

# Supplementary Information: Long-range spatio-temporal correlations in multimode fibers for pulse delivery

Xiong *et al.*

### Supplementary Note 1: Correlations and temporal enhancement

In this section, we review the mathematics behind the known static correlations in disordered media, and then discuss their consequences for the spatio-temporal correlations in multimode fibers. We then introduce our model for temporal control. Finally we discuss the isotropic approximation of the time-resolved transmission matrix.

#### Correlations and transmission eigenvalues

The propagation of monochromatic light through a system with $N$ input and $N$ output channels is described by an $N$-by-$N$ transmission matrix $u$, where the matrix element $u_{ba}$ is the flux-normalized field transmission coefficient from input channel $a$ to transmitted channel $b$. We denote $U_{ba} \equiv |u_{ba}|^2$.

We consider the singular-value decomposition $u = W\sqrt{\tau}V^\dagger$; here $W$ and $V$ are $N$-by-$N$ unitary matrices, and $\tau$ is a diagonal matrix whose elements $\{\tau_n\}_{n=1}^N$ are the eigenvalues of $u^\dagger u$.

For disordered media, the intensity correlation between the transmitted speckles is defined in channel space as

$$C_{aa',bb'} \equiv \frac{\langle U_{ba}U_{b'a'}\rangle}{\langle U_{ba}\rangle\langle U_{b'a'}\rangle} - 1 \tag{1}$$

where $\langle\cdots\rangle$ denotes ensemble average over different disorder realizations. If the matrices $u$ are *isotropic*, the correlations must take on the form [1, 2]

$$C_{aa',bb'} = \delta_{aa'}\delta_{bb'}C_1 + (\delta_{aa'} + \delta_{bb'})C_2 + C_3, \tag{2}$$

where the constants $C_1$, $C_2$, $C_3$ are commonly referred to as the magnitudes of the short-range, long-range, and infinite-range correlations. Typically $C_1 \gg C_2 \gg C_3$.

Mathematically, the transmission matrices $u$ are "isotropic" when the matrices $W$ and $V$ are sampled uniformly and independently from the space of all random unitary matrices in the ensemble average. Physically, isotropy means that all input modes and all output modes are fully mixed, and that all modes are statistically equivalent (a detailed discussion about isotropy of transmission matrix in subsection D).

Spatially, a similar structure emerges for intensity correlation function. Denote $I(\mathbf{r}_b, \mathbf{r}_a)$ as the transmitted intensity at position $\mathbf{r}_b$ on the back surface given a point-source excitation at position $\mathbf{r}_a$ on the front surface. For isotropic disordered media, the correlation between $I(\mathbf{r}_b, \mathbf{r}_a)$ and $I(\mathbf{r}_{b'}, \mathbf{r}_{a'})$ takes on the form [3, 4]

$$\begin{aligned} C(\mathbf{r}_{a'} - \mathbf{r}_a, \mathbf{r}_{b'} - \mathbf{r}_b) = {} & [F(\mathbf{r}_{a'} - \mathbf{r}_a)F(\mathbf{r}_{b'} - \mathbf{r}_b)]C_1 \\ & + [F(\mathbf{r}_{a'} - \mathbf{r}_a) + F(\mathbf{r}_{b'} - \mathbf{r}_b)]C_2 + C_3, \end{aligned} \tag{3}$$

where the coefficients $C_1$, $C_2$ and $C_3$ are the same as in Eq. (2). $F(\mathbf{\Delta r})$ is a function that decays from one when $\mathbf{\Delta r} = 0$ to zero when $|\mathbf{\Delta r}|$ is large. Given a fixed input such that $\mathbf{r}_{a'} = \mathbf{r}_a$, we obtain Eq. (1) in the main text, where we define $\tilde{C}_1 \equiv C_1 + C_2 \approx C_1$ and $\tilde{C}_2 \equiv C_2 + C_3 \approx C_2$.

The statistics of the transmission eigenvalues uniquely determines the magnitudes of the correlations. It was shown rigorously that [1, 2]

$$\begin{aligned} C_1 &= \frac{N^2(N^2+1)}{(N^2-1)^2}\left(\frac{\langle\alpha^2\rangle}{\langle\alpha\rangle^2} - \frac{2N}{N^2+1}\frac{\langle\alpha_2\rangle}{\langle\alpha\rangle^2}\right), \\ C_2 &= \frac{N^2(N^2+1)}{(N^2-1)^2}\left(\frac{\langle\alpha_2\rangle}{\langle\alpha\rangle^2} - \frac{2N}{N^2+1}\frac{\langle\alpha^2\rangle}{\langle\alpha\rangle^2}\right), \\ C_3 &= C_1 - 1, \end{aligned} \tag{4}$$

where $\alpha \equiv \sum_{n=1}^N \tau_n$ and $\alpha_2 \equiv \sum_{n=1}^N \tau_n^2$.

Even though the preceding results were initially obtained in the context of monochromatic light propagation through disordered media, the mathematical framework is very general. In fact, Eqs. (2)–(4) are valid as long as the matrices $u$ are isotropic [2, 3]. In particular, the propagation of a pulse through a multimode fiber will also satisfy Eqs. (2)–(4) if the time-resolved transmission matrix $u(t)$ at arrival time $t$ is sufficiently isotropic.

## Effective model for temporal enhancement

As described in the main text, the largest-possible enhancement of the spatially integrated intensity at a target arrival time $t_0$ is given by the largest eigenvalue of $u^\dagger(t_0)u(t_0)$. Eq. (4) relates the spread of eigenvalues to the magnitudes of the correlations. Here, we provide a heuristic model to relate the maximal eigenvalue to the spread of eigenvalues and then to the correlations.

We model the eigenvalues $\{\tau_n(t_0)\}_{n=1}^N$ associated with the $N$-by-$N$ matrix $u(t_0)$ using the eigenvalues $\{\tau_n^{(\text{eff})}(t_0)\}_{n=1}^N$ associated with an effective $N^{(\text{eff})}(t_0)$-by-$N$ matrix $u^{(\text{eff})}(t_0)$ with uncorrelated matrix elements. The idea, as first introduced in Ref. [5], is to capture the effects of correlations in $u(t_0)$ by resizing the number of output channels from $N$ to $N^{(\text{eff})}(t_0)$. As the magnitude of correlations depends on the arrival time (see Fig. 2c in the main text), the effective number $N^{(\text{eff})}(t_0)$ will also depend on $t_0$.

Given a large $M$-by-$N$ random matrix $u$ with uncorrelated elements, the eigenvalues of $u^\dagger u$ are given by the Marčhenko–Pastur distribution [6], with the largest eigenvalue being $\tau_{\max} = (1+\sqrt{N/M})^2\bar{\tau}$ and the eigenvalue variance being $\text{var}(\tau) \equiv \bar{\tau^2} - \bar{\tau}^2 = (N/M)\bar{\tau}^2$. Therefore, the normalized maximal eigenvalue is directly related to the spread of eigenvalues, as

$$\frac{\tau_{\max}}{\bar{\tau}} = \left(1+\sqrt{\frac{\text{var}(\tau)}{\bar{\tau}^2}}\right)^2. \tag{5}$$

Here, we use overhead bars to denote averaging over the $N$ eigenvalues.

The spread of eigenvalues is related to the magnitudes of the correlations through Eq. (4). Specifically, we obtain

$$\frac{\langle\text{var}(\tau)\rangle}{\langle\bar{\tau}\rangle^2} = \left(1-\frac{1}{N^2}\right)(1+NC_2+C_3) \approx 1+NC_2 \tag{6}$$

by writing $\text{var}(\tau) = (\alpha_2/N) - (\alpha^2/N^2)$ and solving for $\langle\alpha_2\rangle/\langle\alpha\rangle^2$ and $\langle\alpha^2\rangle/\langle\alpha\rangle^2$ in Eq. (4).

Inserting Eq. (6) into Eq. (5), we get

$$\eta(t_0) = 2 + NC_2(t_0) + 2\sqrt{1+NC_2(t_0)}, \tag{7}$$

which is Eq. (4) in the main text. This expression concisely relates the long-range spatio-temporal correlations $C_2(t)$ to the maximal power enhancement at a targeted arrival time $t_0$. It has two underlying assumptions, the first being the isotropy of $u(t_0)$ [which underlies Eq. (4) and Eq. (6)], the second being the heuristic modeling of $\{\tau_n\}$ using a resized uncorrelated matrix [which underlies Eq. (5)].

## Model for focused pulse shape

Experimentally, we also measure the long-range correlation between speckle grains at different arrival times. Such information can be used to predict the pulse shape (output power as a function of $t$) when the input wavefront has been optimized to enhance the power at arrival time $t_0$. We construct a heuristic model based on two observations. First, the normalized power $\eta(t,t_0)$ should be identical to Eq. (7) when $t=t_0$. Second, when the correlation $C_2(t,t_0)\approx 0$, the power at $t$ should be equal to that of random incident wavefront, giving $\eta(t,t_0)\approx 1$. To satisfy these two constraints, we propose

$$\begin{aligned}\eta(t,t_0) = {} & 2C_1(t,t_0) + NC_2(t,t_0) \\ & + 2\sqrt{1+NC_2(t,t_0)} - \beta[1-C_1(t,t_0)].\end{aligned} \tag{8}$$

$C_1(t,t_0)=1$ for $t=t_0$, and $C_1(t,t_0)=0$ for $t\neq t_0$. The parameter $\beta$ is chosen such that the temporally-integrated output power (pulse energy) equals that from a random input. From numerical simulations of a multimode waveguide without loss, we find that $\beta\approx 1$. With loss, both numerical and experimental results confirm that Eq. (8) is still an excellent model with $\beta = 1$.

## Isotropy of time-resolved transmission matrix

Macro- and micro-bendings of a multimode fiber (MMF) cause random mode coupling. In the experiment, we clamp the fiber at multiple locations to introduce micro-bendings. The perturbations induce strong mode mixing

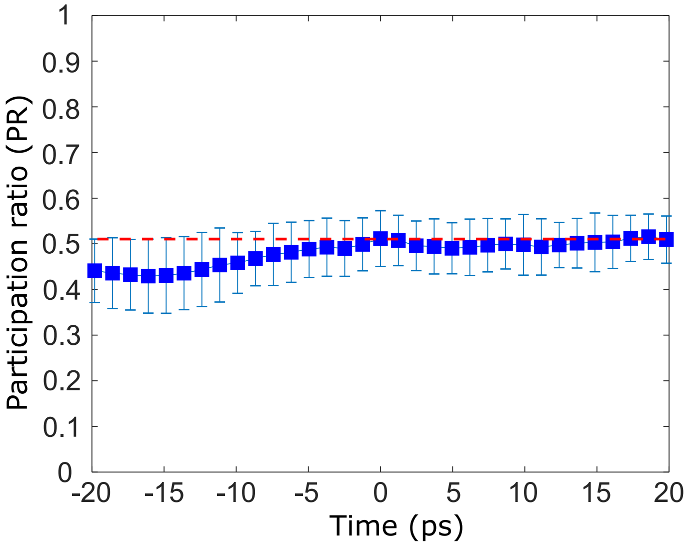


Supplementary Figure 1: Participation ration (PR) of the time-resolved transmission matrices at different arrival times. The error bar is the standard deviation for different input modes at a fixed arrival time. Red dashed line: the PR of isotropic random matrices.

as light propagates through the fiber. In the main text, we present the measured transmission matrices at different arrival times (Fig. 1**c-d**).

At the central (mean) arrival time, the higher-order modes have slightly smaller magnitude than the lower-order modes (Fig. 1d). This is caused by the mode-dependent-loss (MDL) in the fiber. Even with strong mode coupling, the distribution of mode intensities depends on the relative strength of mode coupling and MDL. Since random mode coupling can be regarded as scattering in the fiber mode space, we define the transport mean free path $l_t$ as the propagation length at which light originally injected to a single fiber mode is scattered to all spatial modes. If the fiber length $L \gg l_t$ and $l_t$ is much smaller than the absorption length $l_a$ for any fiber mode, mode mixing dominates over dissipation, then all modes would have similar magnitudes. However, such conditions are not met for the multimode fiber in our experiment. Because the higher-order modes experience significantly more loss, they have shorter $l_a$ than the lower-order modes. As their $l_a$ become shorter than $l_t$, the higher-order modes dissipate faster than mode coupling. Thus their magnitudes become smaller than those of the lower-order modes.

Furthermore, Fig. 1c shows the transmission matrix at early arrival time has more contributions from the lower-order modes than the higher-order modes. In order to have a short delay, the pulse must travel mostly in the lower-order modes that have smaller group delay. Thus the lower-order modes contribute more to the transmission matrix at earlier arrival time. Similarly, the higher-order modes contribute more to the transmission matrix at later arrival times. Therefore, even if the mode-dependent loss is negligible, the transmission matrices at very early or very late arrival times are not expected to be isotropic.

Clearly "isotropy" is an approximation for the time-resolved transmission matrices. To check how good the approximation is, we compute the participation ratio $\mathrm{PR} = \frac{(\sum_{i=1}^{N} I_i)^2}{N\sum_{i=1}^{N} I_i^2}$, where $I_i$ is the transmitted intensity in mode $i$. The larger the PR, the more uniform the transmitted light is spread to all modes. For the measured transmission matrix at a fixed delay time, we calculate the PR for light injected to each fiber mode (each column) and average the PR over all input modes (all columns). To quantify the fluctuations of PR for different input modes, we compute the standard deviation of PR for all columns of the transmission matrix.

The mean value and the standard deviation of PR are plotted versus the arrival time in Fig. S1. For comparison, we also compute the PR for isotropic random matrices. Each column of the matrix is normalized to unity and consists of $N$ complex numbers that are randomly chosen from the normal distribution. The ensemble-averaged PR is 0.51. The mean value of PR at different arrival times is fairly close to 0.51 (red dashed line) in Fig. S1. Hence, isotropy is a good approximation for the time-resolved transmission matrices of our fiber within the measurement range of delay times.

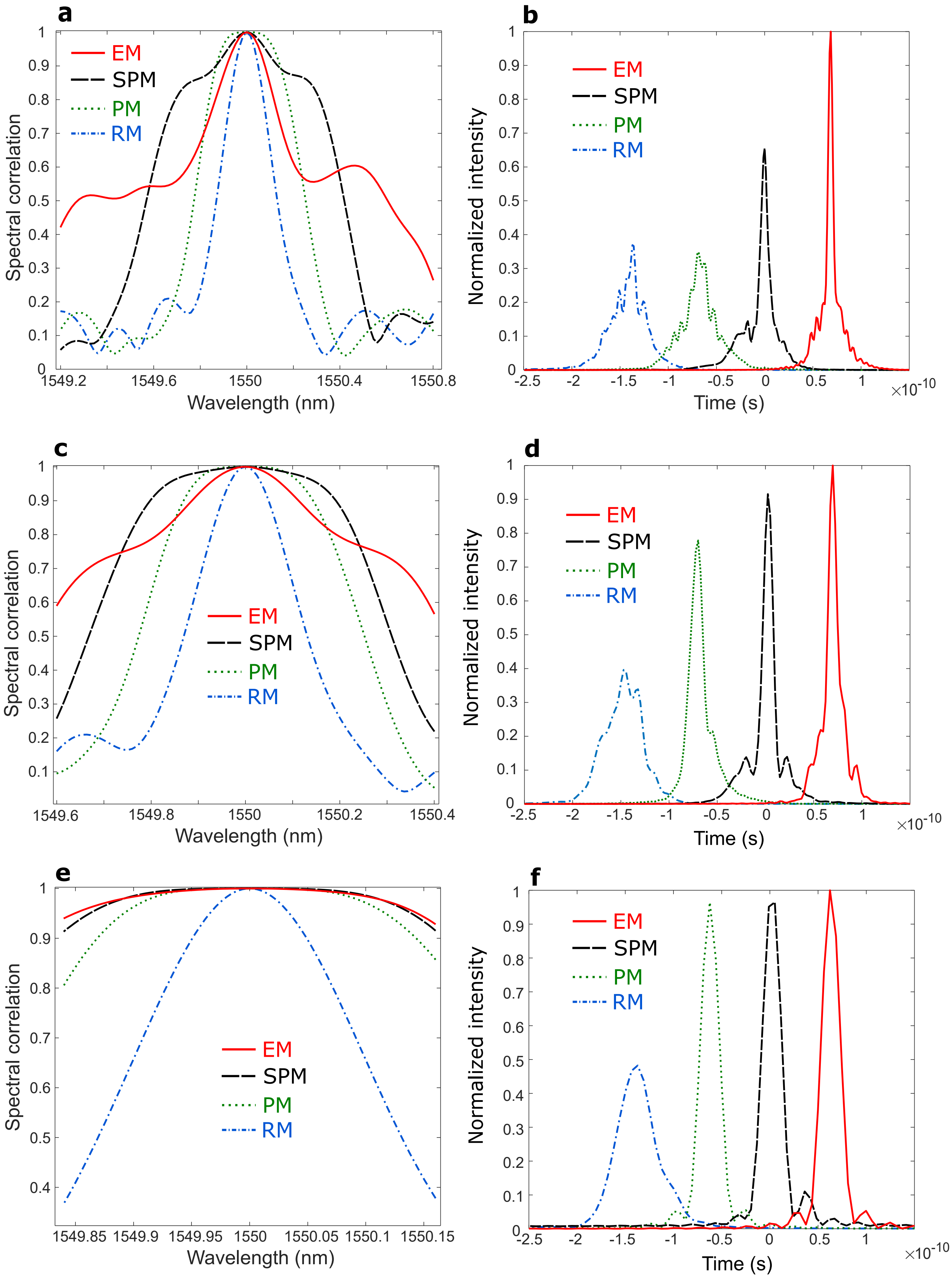


Supplementary Figure 2: Spectral correlation function $C(\Delta\lambda)$ of the output field pattern and the corresponding temporal pulse shape when the input pulse has the spatial wavefront set by an EM, SPM, PM and random wavefront (RM). The input pulse is transform limited and has a Gaussian spectrum of width equal to (a, b) 10, (c, d) 5 and (e, f) 2 times of the spectral correlation width of the multimode waveguide. For clarity, the transmitted pulses are temporally off-set by 70 ps in (b, d, f).

## Supplementary Note 2: Comparison to Principal Modes

The principal mode (PM) is defined as the eigenmode of the group-delay matrix for a multimode fiber [7–10]. It retains the transmitted spatial field profile to the first order of frequency variation. If random mode coupling is strong in a fiber ($L \gg l_t$), the spectral window, over which the transmitted field pattern remains nearly unchanged with frequency, has a width about twice of the spectral correlation width of the fiber. To further enhance spectral correlation, super-PMs [11] are created by minimizing the variation of output field pattern with frequency, i.e., by maximizing the area underneath the curve for the spectral correlation function over a chosen frequency range. The spectral bandwidth of the super-PM is about four times of the spectral correlation width of the fiber with strong mode mixing.

Both PMs and super-PMs aim to achieve an output spatial profile that is invariant in frequency so that the output field pattern does not change with the arrival time and maintains the spatial coherence; however, the transmitted power is not necessarily maximized at any arrival time.

The eigenmode (EM) with the maximum eigenvalue of the time-resolved transmission matrix generates the highest possible transmitted power at the selected arrival time. It works for input pulses with arbitrarily broad spectra. Therefore, PM, super-PM (SPM) and EM are all suppressing modal dispersion in the fiber, but for different purpose and thus optimizing the incident wavefront with different figures of merit.

Below we compare the spectral correlation and temporal pulse shape of the PM, super-PM (SPM), EM and random wavefront (RM) for varying bandwidths of the input pulse. Numerically we simulate a multimode waveguide with the concatenated model [12]. The input pulse is assumed to have a Gaussian spectrum. Its spectral width is varied from 2, 5 and 10 times of the correlation width of the fiber (for random incident wavefronts). The spectral correlation function for the transmitted field pattern $|\psi_{out}\rangle$ is given by $C(\Delta\lambda \equiv \lambda - \lambda_0) \equiv \langle\psi_{out}(\lambda_0)|\psi_{out}(\lambda)\rangle$, where $|\psi_{out}\rangle$ is normalized at each wavelength $\lambda$ [9].

Figure 2 presents the results for three bandwidths of input pulses, which are transform limited. For the EM, $t_0$ is set to the mean arrival time. The PM and SPM are chosen to have the intermediate delay time and the broadest bandwidth. When the input bandwidth is 10 times of the spectral correlation width of the fiber (as studied in our experiment), EM clearly outperforms PM and SPM in both the frequency domain and the time domain (**a,b**). The total area underneath the curve of the spectral correlation function for EM is the largest, indicating the overall spectral decorrelation within the broad range is the least for EM (**a**). Temporally, EM achieves the highest peak power of the transmitted pulse (**b**). When the input bandwidth is reduced to 5 times of the spectral correlation width (**c,d**), the PM still displays notable spectral decorrelation and the output pulse is clearly broadened in time. The SPM and EM are effective in suppressing spectral decorrelation (**c**) and temporal stretching (**d**), but EM still outperforms SPM slightly. Only when the input spectrum is reduced to twice of the spectral correlation width of the fiber (**e,f**), PM, SPM and EM achieve almost equivalent performances. In the frequency domain, PM, SPM and EM barely decorrelate, contrary to RM (**e**). In the time domain, the output pulse for RM is about twice longer than the input pulse. In contrast, PM, SPM and EM all have transmitted pulses of length comparable to the incident pulse (**f**).

## Supplementary References


[1] Mello, P. A., Akkermans, E. & Shapiro, B. Macroscopic approach to correlations in the electronic transmission and reflection from disordered conductors. *Phys. Rev. Lett.* **61**, 459 (1988).

[2] Mello, P. A. Averages on the unitary group and applications to the problem of disordered conductors. *J. Phys. A* **23**, 4061 (1990).

[3] Cwilich, G., Froufe-Pérez, L. S. & Sáenz, J. J. Spatial wave intensity correlations in quasi-one-dimensional wires. *Phys. Rev. E* **74**, 045603 (2006).

[4] Yamilov, A. Relation between channel and spatial mesoscopic correlations in volume-disordered waveguides. *Phys. Rev. B* **78**, 045104 (2008).

[5] Hsu, C. W., Liew, S. F., Goetschy, A., Cao, H. & Stone, A. D. Correlation-enhanced control of wave focusing in disordered media. *Nat. Phys.* **13**, 497-502 (2017).

[6] Marčhenko, V. A. & Pastur, L. A. Distribution of eigenvalues for some sets of random matrices. *Math. USSR Sb*, **1**, 457 (1967).

[7] Fan, S., & Kahn, J. M. Principal modes in multimode waveguides. *Opt. Lett.* **30**, 135-137 (2005).

[8] Carpenter, J., Eggleton, B. J. & Schröder, J. Observation of Eisenbud–Wigner–Smith states as principal modes in multimode fibre. *Nat. Photon.* **9**, 751 (2015).

[9] Xiong, W. *et al.* Spatiotemporal control of light transmission through a multimode fiber with strong mode coupling. *Phys. Rev. Lett.* **117**, 053901 (2016).

[10] Xiong, W. *et al.* Principal modes in multimode fibers: exploring the crossover from weak to strong mode coupling. *Opt. Express*, **25**, 2709-2724 (2017).

[11] Ambichl, P. *et al.* Super-and Anti-Principal-Modes in Multimode Waveguides. *Phys. Rev. X* **7**, 041053 (2017).

[12] Ho, K. P., & Kahn, J. M. Statistics of group delays in multimode fiber with strong mode coupling. *J. Light. Technol* **29**, 3119-3128 (2011).
